# Incidence of Varus Malalignment Post Interlocking Nail in Proximal Femur Shaft Fractures Comparing Two Types of Entry Points

**DOI:** 10.5704/MOJ.1711.013

**Published:** 2017-11

**Authors:** AN Sadagatullah, MN Nazeeb, S Ibrahim

**Affiliations:** Department of Orthopaedics, Universiti Sains Malaysia, Kubang Kerian, Malaysia; ^*^Department of Orthopaedics, Hospital Sultan Ismail, Johor Bahru, Malaysia

**Keywords:** interlocking nail, greater trochanter entry point, varus deformity, femur shaft fracture

## Abstract

**Introduction:** Osteosynthesis of the femur using an interlocking nail is the gold standard for treating diaphyseal fractures of the femur. There are two established entry points for the antegrade interlocking nails which is the piriformis fossa or the greater trochanter. It has been reported that varus malalignment was frequently seen in proximal femur fracture which were treated with interlocking nail utilizing the greater trochanter entry point. The study was done to find out if the problem was of significance.

**Materials and Methods:** This was a retrospective study which included 179 patients with femur fractures which were treated from January 2013 till September 2015 in one Hospital. They were treated with interlocking nail either by utilizing the piriformis fossa (PF) or the greater trochanter (GT) entry points. Post-operative radiographs of the femur were used to measure the varus deformity.

**Results:** Out of 179 patients, there were 5 patients who were reported to have unacceptable varus malalignment (2.79%). These 5 patients were out of the 88 (5.68%) patients utilizing the greater trochanter as the entry point. The same 5 patients were out 90 patients that were diagnosed with proximal femur shaft fractures (5.55%). Analysis with logistic regression was statistically not significant.

**Conclusion:** There was higher rate of varus malalignment seen in proximal femur shaft fractures treated with interlocking nails utilizing the greater trochanter entry point. The incidence of varus malalignment was not significant statistically.

## Introduction

Femur is the principal weight bearing bone of the lower extremity and fracture of femur leads to considerable morbidity and mortality. Femoral shaft fracture usually results from high energy trauma which may be associated with multisystem. Early fixation of femur shaft fracture may prevent grave complications like fat embolism and acute respiratory distress syndrome^1^. It also allows early mobilization, reducing the risks of hip and knee stiffness as well as quadriceps and hamstring wasting.

Osteosynthesis of the femur using an intramedullary nail is considered to be the gold standard for treating diaphyseal fractures of the femur. This is considered to be superior to extramedullary fixation using plates and external fixators, from both the biomechanical and the clinical points of view^2^. Intramedullary nail provides predictable restoration of shaft length and alignment and allowed early load bearing. The piriformis fossa and greater trochanter has been commonly described as starting points for ante grade femoral nailing. As the greater trochanter is not collinear with the long axis of the femoral shaft, complications including varus malalignment and iatrogenic fracture comminution have been demonstrated to occur when nails designed for insertion through the piriformis fossa are inserted through the greater trochanter^3^. However, the favoured entry point for nails has been debated with advocates for both the piriformis fossa and greater trochanteric entry points^4,5^. The greater trochanteric entry point is technically easier due to the subcutaneous location of the greater trochanter, especially in obese patients^3^. Furthermore, it is less sensitive to anteroposterior translation due to the more cancellous nature of the trochanteric area and it reduces risk of iatrogenic bursting of the proximal segment^4^. It also reduces the risk of damaging the blood supply to the femoral head because of its more lateral approach^4^. Its potential disadvantages are iatrogenic fracture of the greater trochanter and varus malalignement^5^.

Nails inserted through the greater trochanter have been shown to have equal rates of union, complication, and functional outcome compared to those inserted through the piriformis fossa^3^. Trochanteric nails were superior in decreasing the fluoroscopy and surgery time in obese patients, there are less destructive to the abductor musculature and there is less blood loss^3^. There were reported cases of varus malalignment in trochanteric nails occurring in proximal femoral shaft fractures. However, the data is insufficient in comparing these two entry points with regards to a varus malalignment. Proximal femoral shaft fractures should be given extra consideration because of the difficulties encountered in its treatment, which are related to the anatomic and biomechanical features unique to this area.

The purpose of this study was to compare the incidence of varus malalignment in proximal femoral shaft fracture which were surgically treated with interlocking nails through the greater trochanter and through the piriformis fossa. In this study varus deformity was considered unacceptable when there is a varus angulation of more than 10 degrees at the fracture site on an antero-posterior radiograph as defined by Kraemer *et al*^6^. Only these cases were reported as malalignment.

## Materials and Methods

**Fig. 1:**
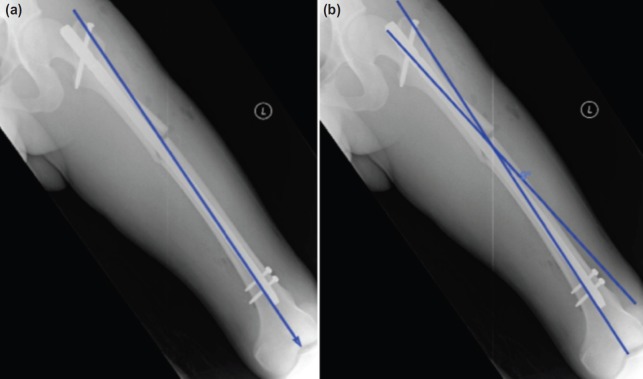
(a) A first line is drawn along the anatomical axis of the distal fragment of the femur and (b) A second line is drawn along the anatomical axis of the proximal segment of the femur and the intersecting angle between these two lines is the varus angle.

In this retrospective study, 179 patients in Hospital Sultan Ismail, Johor Bahru were enrolled from January 2013 till September 2015. These patients had either a proximal shaft or midshaft femur fracture and were surgically treated with an interlocking nail. The inclusion criteria were patients aged between 16-60 years old who underwent ante grade interlocking nailing for proximal shaft and midshaft femur fractures either through the piriformis fossa (PF) and greater trochanter (GT) as entry points. The two types of interlocking nails which were used in this study were the Synthes Shaft Femoral Nail (SSFN) by Synthes® which designed for greater trochanter as its entry point, and the Aesculap® Targon F/T nail by B. BRAUN® which utilizes the piriformis fossa as its entry point. Patients with a distal third shaft femur fracture, inter trochanteric fracture, pathological fracture and patients who had ante grade nailing following open reduction technique were excluded from this study. Immediate postoperative anterior posterior radiographs of the operated femur was acquired and the measurement of fracture alignment on the radiographs were done using Centricity® Radiology Web version 1.0 2002 by GE Medical System. Anatomical axes of each bone segment can be defined with a line drawn through the centre of the diameter of the diaphysis of each bone segment at two levels. The angle between the proximal and distal anatomical axes will depict the degree of angulation^7^ (Fig. 1).

All data were entered into International Business Machines (IBM) ® Statistical Packages for Social Sciences (SPSS) version 22.0 licensed to our institution. For the univariate analysis, p-value obtained from the independent t-test with level of significance <0.05. Independent t-test was used to determine the potential mean difference with an outcome. Assumptions of a random sample were made by Levene`s test. For the descriptive analysis, numerical variables were described as mean and standard deviations. Categorical data were presented in frequency and percentage. Each categorical data was also analysed by logistic regression to ascertain the difference between the data.

## Results

The profile of patients in the study were shown in Table I. A total of 179 patients were included within the study period out of which 161(89.9%) were male and 18(10.1%) were female with a mean age of 29 years old. There were 88 (49.2%) fractures treated with SSFN using greater trochanter (GT) as the entry point, and 91(50.8%) fractures treated with Targon nails with piriformis fossa (PF) as the entry point. The mean angulation between the fracture segments was 3.43 degrees. Five out of 88 patients (2.8%) from the GT group had varus malalignment of more than 10º, while there no malalignment was noted in the PF group. It was found that the mean varus malalignment between GT group and the PF group was statistically significant (p<0.001), 95% CI of mean difference: 0.74, 2.30 (Table II).

**Table I:** Profile of patients

Variable	Mean (SD)	Frequency (%) (N=179)
Age	29.15(10.20)	
Varus deformity angle	3.43(2.75)	
Sex		
Male		161(89.9)
Female		18(10.1)
Implant		
Synthes		88(49.2)
Targon		91(50.8)
Entry point		
GT		88(49.2)
PF		91(50.8)
Varus deformity		
Yes	13.6°	5(2.8)
No		174(97.2)
Diagnosis		
Proximal		90(50.3)
Midshaft		89(49.7)

**Table II:** Mean difference of varus deformity comparing GT and PF entry points

Variable (mm)	GT, Mean (SD)	PF Mean (SD)	Mean difference (95% CI)	t statistics (df)	p value (SD)
Varus deformity	4.20(3.16)	2.68(2.03)	1.52(0.74, 2.30)	3.84(177)	<0.001

The degree of angulation measured was grouped into three to demonstrate the measured incidence of varus angulation (Table III). Most of the patients in PF group had varus angulation less than 4.9 degrees. In the GT group, we noted higher incidence of varus angulation of more than 5 degrees (13.4 percent). However, only 2.8 percent of all the cases (N=179) were noted to have varus angulation of 10 degrees or more.

**Table III:** Measured varus angle based on entry points

Varus Angle / Entry point		Number of patients (% of patients)			Total patients, N (%)		
		0º to 4.9º	5º to 9.9º	>10º			
**GT**		64(35.8)	19(10.6)	5(2.8)		88(49.2)	
	**PF**		84(46.9)	7(3.9)	0(0)		91(50.8)
							179(100)

GT=Greater Tochanter

PF= Piriformis Fossa

**Fig. 2:**
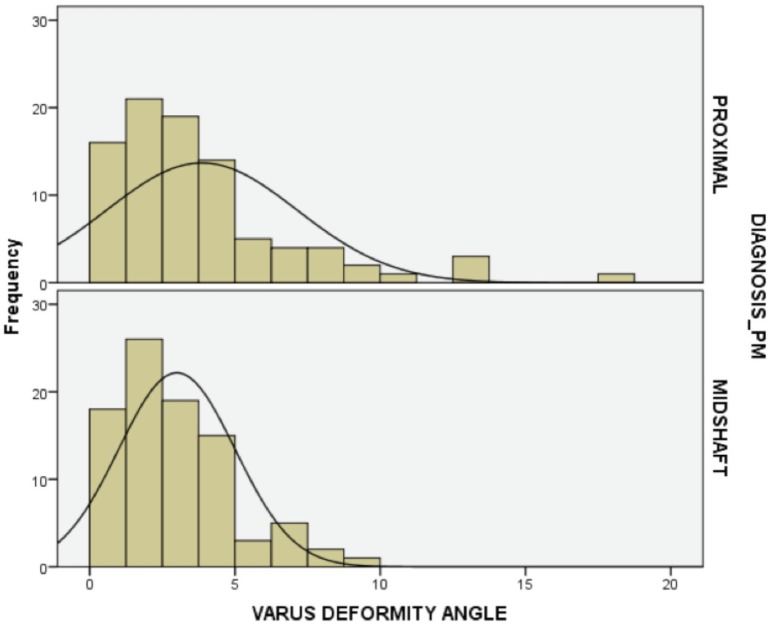
Frequency histogram of the cases treated according to site of fracture (either proximal femoral fractures (Proximal) or midshaft femoral fractures (Midshaft).

There were 89 patients with midshaft fractures and 90 patients with proximal femur fractures. Five of the proximal femur fracture group had varus malalignment with a mean varus angulation of 13.6 degrees. There was no malalignment noted in the midshaft femur fracture group (Fig. 2). Despite the five fractures with more than 10 degrees of varus angulation in the proximal femur fracture group, logistic regression analysis showed that the association (with the proximal femur fracture as an independent variable with varus as outcome) was not statistically significant.

## Discussion

Surgical intervention is the mainstay of treatment for femur shaft fractures. Non-operative management is seldom chosen due to issues with limb shortening, mal-rotation, as well as the high morbidity and mortality rates secondary to prolonged recumbence associated with skeletal traction, especially in the elderly. It is also well known that the proximal femur fractures are the most difficult amongst the femur shaft fractures to treat because of the intraoperative difficulty in reduction. In this study, we have shown that some degree of varus malalignment is common in proximal femur fractures which were treated with interlocking nails using the greater trochanter as its entry point.

Varus malalignment over the proximal femur commonly occurs because of the actions of the surrounding musculature^1,6,8,9^. The proximal fragment will be abducted due to the actions of gluteus medius and minimus and the fragment will also be flexed due to the insertion of illiopsoas. The action of the adductor muscles usually causes adduction, medialization and shortening of the distal femur and this overall makes intraoperative reduction ever so difficult in cases of proximal femur shaft fractures.

The original starting point advocated by Kuntscher for femoral nailing was located on the lateral aspect of the greater trochanter^5,10^. Winquist *et al* noted that this led to fracture site comminution at the medial femoral cortex^11-13^. In his series Winquist subsequently chose the piriformis fossa as the entry point for femoral nailing. The piriformis fossa entry point continues to be used although lateral bend nails have been specifically developed to allow entry through the tip of the greater trochanter. Ricci *et al* found no difference in union rates or complications with the use a lateral entry femoral nail^3^.

Biomechanical studies have shown that a more lateral entry point leads to varus malreduction, which would be related to increase bending stresses^14^. The use of the greater trochanter entry point also accounts for the higher strain levels seen lateral to the insertion point. Previous clinical series described iatrogenic fracture, varus malalignment, and comminution at the medial cortex when a straight nail is inserted through the greater trochanter. Fractures were often associated with a more anteriorly located starting hole^15,16^. Even though the greater trochanter entry point is technically easier to perform because of its subcutaneous location, it has a higher risk of mal reduction (varus) because it’s not collinear along the axis of the femur unlike the piriformis fossa entry point^4,17^.

In this study, we showed that nailing with greater trochanter entry point had higher incidence rate of varus malaignment compared to nailing with piriformis fossa entry point, and this was also reported by Ricci *et al*^3^ and Yun *et al*^4^. It was also found that proximal femur shaft fractures presented with higher postoperative varus malalignment after fixation with a interlocking nail compared to midshaft fractures and the results were almost identical with Geogiadris *et al*^18^.

Varus angulation disturbs the normal transmission of force across the knee, and altered stress distribution related to deformity has been shown in cadaver models using pressure-sensitive film. This would eventually lead to degenerative arthropathy^12^. Therefore, it is of clinical importance that we consider the location of the femoral shaft fractures is determined before choosing the type of interlocking nail along with its corresponding entry sites.

The study has avenue for many improvements. We are using a single nail design for the trochanteric entry nail model. Therefore it is possible that the findings may be related to the design of the nail and not actually related to the technique. The sample cases for analysis was from a single centre done in retrospect, with the final number too small to a get conclusion. A prospective and randomized multicentre study with a larger number of patients would make the study better and more meaningful. More variables like the patients’ weight or body mass index maybe relevant to reflect the difficulty of case selection. The study measured only immediate post operative radiographs; a longer follow up study to look at remodelling potential and varus angle after the patient became ambulant would assist in assessment of the detriments of having a varus deformed femur. A longer follow up was however beyond the scope of the study.

## Conclusion

There was a significantly higher incidence of varus malalignment of more than 10 degrees for fixation of proximal and midshaft femur fractures treated with interlocking nails designed for greater trochanter entry point. Although all of them were noted in proximal femur fractures, the association were not statistically significant.
